# Validity and Reliability of the Persian Versions of National Institute of Health Stroke Scale and Modified National Institute of Health Stroke Scale in Hospitalized Patients

**DOI:** 10.31661/gmj.v8i0.1188

**Published:** 2019-01-25

**Authors:** Reza Dehghani, Afshin Borhanihaghighi, Abdolhamid Shariat, Mohammad Nami, Masoume Nazeri, Amin Abolhasani Foroughi, Samrad Mehrabi, Masoumeh Emamghoreishi

**Affiliations:** ^1^Clinical Neurology Research Center, Shiraz University of Medical Sciences, Shiraz, Iran; ^2^Department of Pharmacology, School of medicine, Shiraz University of Medical Sciences, Shiraz, Iran; ^3^Department of Neurology, School of medicine, Shiraz University of Medical Sciences, Shiraz, Iran; ^4^Department of Neuroscience, School of Advanced Medical Sciences and Technologies, Shiraz University of Medical Sciences, Shiraz, Iran; ^5^Department of Radiology, School of medicine, Shiraz University of Medical Sciences, Shiraz, Iran; ^6^Sleep Disorders Laboratory, Division of Pulmonology, Department of Internal Medicine, Shiraz University of Medical Sciences, Shiraz, Iran

**Keywords:** National Institute of Health Stroke Scale, Modified National Institute of Health Stroke Scale, Persian Version, Stroke

## Abstract

**Background:**

National Institute of Health Stroke Scale (NIHSS) and Modified National Institute of Health Stroke Scale (mNIHSS) are two valid and reliable questionnaires that assess stroke severity. This study aimed to examine and compare the validity and reliability of Persian versions of NIHSS and mNIHSS in hospitalized patients.

**Materials and Methods:**

The English versions of NIHSS and mNIHSS were translated to Persian (forward and backward), and three neurologists examined the face and content validity of both questionnaires. The Persian versions of NIHSS and mNIHSS were used in 75 hospitalized stroke patients (hemorrhagic and obstructive) admitted to Namazi teaching hospital, Shiraz, Iran. The reliability and validity of the Persian versions were examined by Cronbach’s alpha coefficient and convergent validity.

**Results:**

The values of Cronbach’s alpha for Persian versions of NIHSS and mNIHSS were 0.81 and 0.86, respectively. The scaling success of convergent validity in NIHSS and mNIHSS were 80% and 100%, respectively.

**Conclusion:**

The Persian versions of NIHSS and mNIHSS were reliable and valid. However, mNIHSS was more valid and reliable than NIHSS. Persian version of mNIHSS can be suggested to be used for assessing stroke severity in hospitalized stroke patients by neurologists and researchers.

## Introduction

National Institute of Health Stroke Scale (NIHSS) is a ubiquitous, valid, and reliable clinical questionnaire predicting the severity of stroke. This questionnaire is useful for making medical and nursing decisions and is routinely being used in clinical trials [1-5]. It has 15 items including the level of consciousness (LOC), LOC questions, LOC commands, best gaze, visual, facial palsy, motor arm, motor leg, limb ataxia, sensory, best language, dysarthria, extinction, and inattention. Higher scores on the scale is an indicator of more severe neurological deficits [2]. In spite of its reliability, validity and widespread use, the NIHSS has been scrutinized for some redundant items including the OLC, facial palsy, limb ataxia, and dysarthria. These items have caused complexity for NIHSS implementation, prolongation of the questionnaire response time, tiredness of the person filling out the questionnaire and also deviance in its ultimate results. These limitations of NIHSS questionnaire have led to a reduced tendency for implementing and reporting NIHSS in emergency and stroke wards [6, 7], while an initial estimation of stroke severity is essential for the appraisal of the treatment and rehabilitation programs.Modified National Institute of Health Stroke Scale (mNIHSS) is a succeeding questionnaire in which the redundant items have been removed. Such changes have brought several advantages for mNIHSS over NIHSS including simpler grading scale, more accessible to administration, more precise results, and shorter time to perform. These advantages have made the mNIHSS more valid, reliable and acceptable among clinicians and researchers [6, 8].

Stroke is the main cause for physical disability and the second cause of mortality worldwide [9]. Two-thirds of all strokes occur in developing countries including Iran [9, 10]. The incidence of stroke in Iran is about 750-800 individuals in 100000 population per year, which is much higher than worldwide [11, 12]. Thus, having a valid and reliable questionnaire for evaluating the severity of stroke in stroke and emergency wards and clinical trials is an essential need. English version of NIHSS has been routinely used as a reliable and valid questionnaire in Iran. However, since misinterpretation/misunderstanding of the English version might lead to a deviation in the ultimate results, developing a Persian version of NIHSS seems necessary. There is only one previous study assessing the reliability and validity of the Persian version of NIHSS [13]. However, there were some limitations in the preceding study such as evaluation of validity and reliability of the questionnaire in non-hospitalized subjects; inclusion of only ischemic stroke patients; and exclusion of patients who have sever consciousness deficit, severe hearing or vision impairments [13]. Since this questionnaire has been developed to assess the severity of stroke at the time of evaluation in all stroke patients (ischemic or hemorrhagic) regardless of patients’ conditions (such as vision and hearing abilities of patients). Therefore, for assessing the validity of Persian version of the questionnaire all stroke patients has to be included in the study in order to generalize its applicability. Also, the evaluation of the severity of the stroke is needed at the time of admission to hospital in order to follow the improvement of patients’ complications during drug therapy and rehabilitation programs. Thus, it is also necessary to validate this questionnaire in hospitalized stroke patients.Furthermore, considering the advantages of mNIHSS over NIHSS, it is valuable and worthwhile to validate a Persian version of mNIHSS as well. The availability of a Persian version of this easier and less time-consuming questionnaire will encourage medical staffs to use it more frequently at the emergency and stroke wards. Therefore, this study aimed to examine and compare the validity and reliability of Persian versions of NIHSS and mNIHSS in hospitalized stroke patients.

## Materials and Methods

This study was part of a comprehensive research approved by the local Ethics Committee and research council of Shiraz University of Medical Sciences (approval code: 10400). Patients were only enrolled after giving written informed consent. In the case that the patients couldn’t fill the informed consent, their legal guardians signed the consent forms. All procedures were followed in accordance with the guidance and approval of the Institutional Medical Ethics Committee.

### Instruments and Participants

After obtaining permission from the National Institute of Neurological Disorders and Stroke (https://www.ninds.nih.gov), both questionnaires were translated to Persian by a single professional translator (forward translation) and then the translated questionnaires were translated into English (backward translation) by another single native English translator. Three neurologists examined the face and content validity of both translations. The final translated versions were finalized after the agreement of translators and neurologists on items that were difficult to be easily perceived.

Seventy-five patients (39 men and 36 females) aged 27 to 65 years were randomly and sequentially entered the study in order of their admission time to the stroke ward of Namazi teaching hospital, Shiraz, Iran from April 2017 through July 2017. An inclusion criterion was clinical diagnosis of stroke (hemorrhagic or obstructive) by computed tomography scan or magnetic resonance imaging. Patients with previous neurological disorders such as Alzheimer disease, Parkinson or seizure were excluded from the study. Sample size was calculated according to rule of thumb stating that the required number of subjects for testing validity and reliability of a questionnaire equals to at least 5-10 times of number of questions (items) in a questionnaire (i.e., 15 questions in the NIHSS× 5 = 75 subjects is needed for testing validity and reliability of NIHSS questionnaire) [14, 15]. For each patient, both NIHSS and mNIHSS were performed by a single trained researcher. Both NIHSS and mNIHSS questionnaires have multiple Likert-type scale questions. The Likert-type scale questions in both NIHSS and mNIHSS may have three, four or five options that the implementer must choose an answer among the choices for each question. The Likert-type scale questions in both questionnaires determine the patient’s condition in several domains (Table-1). These questionnaires have some differences as well. NIHSS has 15 questions while mNIHSS has 11 questions. In mNIHSS, the LOC, facial palsy, limb ataxia, and dysarthria items have been deleted. Also, question 8 (sensory item), which was a three-option question in NIHSS has been changed to a two-option question in mNIHSS.

### Data Analysis

The collected data from patients were inserted in the SPSS (version 16, IBM USA) and were analyzed for reliability and validity. Reliability and validity of the two questionnaires were assessed by calculating Cronbach’s alpha coefficient and convergent validity of each questionnaire; respectively. Cronbach’s alpha coefficient measures the internal consistency (reliability) and ranges from 0 to 1. The values >0.65 are acceptable [16]. The higher grades (>0.65) indicates more reliable the questionnaire. Convergent validity is a subtype of construct validity. For calculating the convergent validity, the Pearson correlation coefficient was used. A value of a correlation coefficient of greater than 0.40 between an item and total score is regarded as adequate evidence of convergent validity [17]. Also, the scaling success of convergent validity for each questionnaire was calculated by dividing the number of correlations above 0.40 by the total number of correlations ×100 (Table-2).

## Results

Face and content validity are subjective measures. If specialists approve the face and content validity, the questionnaire was applicable for assessing the desired goals. In this study, the face and content validities of Persian versions of NIHSS and mNIHSS questionnaires were approved by three neurologists. Cronbach’s alpha coefficient is the most frequent measure for assessing reliability (internal consistency). It is mostly used when there are multiple Likert-type scale questions in a questionnaire (such as the NIHSS questionnaire) that form a scale, and one wants to specify if the scale is reliable. In this study, Cronbach’s alpha coefficients were 0.81 for NIHSS and 0.86 for mNIHSS (Table-2).

Convergent validity is a subtype of construct validity, which was used to determine whether the NIHSS and mNIHSS were really assessing the severity of stroke. A correlation coefficient of greater than 0.40 is an index of convergent validity. The numbers of questions that have correlation coefficients of greater than 0.4 were 12 in NIHSS and 11 in mNIHSS. This means that the scaling success was 80% and 100% in NIHSS and mNIHSS, respectively (Table-2).

## Discussion

The results of the current study confirmed the reliability and validity of Persian versions of NIHSS and mNIHSS in hospitalized stroke patients. Also, the findings of this study indicated that the Persian version of mNIHSS was more valid and reliable than the Persian version of NIHSS. English version of NIHSS is a valid and reliable questionnaire, which is routinely used to assess the severity of the stroke. In the present study, the Persian version of NIHSS showed an excellent Cronbach’s alpha coefficient confirming its reliability to predict the severity of stroke in hospitalized stroke (ischemic and hemorrhagic) patients. There was only one previous study evaluating the reliability of the Persian version of NIHSS in non-hospitalized ischemic stroke patients [13]. In line with our finding, the Cronbach’s alpha coefficient of the previous study (0.89) also indicated the reliability of Persian version of NIHSS in non-hospitalized stroke patients. Also, the finding of a scaling success of 80% in the present study demonstrated the validity of Persian version NIHSS. This is inconsonant with the results of the previous study using a different method for validity assessment of Persian version NIHSS [13].

The agreements between the outcomes of the current and previous research indicate that the Persian version of NIHSS can be implemented in both hospitalized and non-hospitalized stroke patients. Furthermore, the Cronbach’s alpha coefficient obtained in this study (0.81) was similar to Cronbach’s alpha coefficient reported by Kasner *et al*., for the English version of NIHSS (0.82) [3]. It suggests that the reliability of the current Persian version of NIHSS is comparable to that of the English version. Despite various methods used, the validity and reliability of NIHSS have also been reported in other languages such as Chinese, Italian, Spanish, Estonian, German, Hindi, Hungarian, Marathi, Telugu, and Portuguese [18-22]. mNIHSS, a modified form of NIHSS, is more convenient for use in emergency and stroke wards in clinical settings due to its several advantages over NIHSS. The current study, for the first time, reports the validity and reliability of the Persian version of mNIHSS for the appraisal of stroke severity in hospitalized patients. No other study has yet evaluated the validity and reliability of the Persian version of mNIHSS to compare with our results. However, our findings are in agreement with studies evaluated the validity and reliability of English version of mNIHSS. In the present study, both Persian versions of NIHSS and mNIHSS showed excellent Cronbach’s alpha coefficients representing that both questionnaires have appropriate reliability (internal consistency). These results indicated that the questions in both Persian versions of NIHSS and mNIHSS were generally consistent as a group of items to assess the severity of stroke. Nevertheless, the higher value of Cronbach’s alpha coefficient for mNIHSS revealed that the mNIHSS questions were more consistent and reliable than those of NIHSS. Comparing the validity of two Persian questionnaire, it was demonstrated that mNIHSS was more valid than NIHSS. All questions in mNIHSS had the correlation coefficient of greater than 0.4 leading to a scaling success of 100%. Though, 3 out of 15 questions in NIHSS showed a correlation coefficient of less than 0.40 directing to a scaling success of 80% for Persian version of NIHSS. Interestingly, these three questions including facial palsy, limb ataxia, and dysarthria were those that have been removed from NIHSS to generate mNIHSS. These results are in agreement with previous studies proposing that these questions are redundant and should be removed from NIHSS. In addition, correlation range of convergent validity (0.49-0.79) in mNIHSS was not scattered as much as in NIHSS (0.29-0.83), which represents more coherence among questions of mNIHSS compare to NIHSS. These results demonstrated that mNIHSS was more valid than NIHSS Persian version. The findings of this study are in line with previous studies indicating that the English version of mNIHSS is more valid and reliable than NIHSS [6, 7, 23, 24]. Considering that mNIHSS is easier to implement, needs shorter time to execute and has fewer redundant items, the valid and reliable Persian version of mNIHSS can resolve the discontent with NIHSS and helps nurses, clinicians and researchers to have an easy, suitable and applicable questionnaire to assess the severity of stroke in stroke and emergency wards as well as clinical trials. Replacing NIHSS with mNIHSS in stroke and emergency wards may increase the tendency for reporting the severity of stroke, which is necessary for clinical and nursing decisions as well as in treatment and rehabilitation programs. The limitation of this study was not able to implement the questionnaires with more than one researcher that prevented the calculation of the kappa coefficient (inter-rater agreement). Hence, future study should address this issue.

## Conclusion

Persian versions of NIHSS and mNIHSS were reliable and valid questionnaires. However, the mNIHSS was more valid and reliable questionnaire than NIHSS. With regards to superiorities of mNIHSS vs. NIHSS, replacing NIHSS with mNIHSS in stroke and emergency wards and clinical trials may be an appropriate move.

## Acknowledgment

This study was based on work done by Reza Dehghani in partial fulfillment of the requirements for his Ph.D. thesis in Pharmacology. This study was supported by a grant from vice chancellor for research affair Shiraz University of Medical Sciences (grant No. 10400). The authors appreciate the kind and helpful assistance of nurses in the stroke ward of Namazi teaching hospital, Ms.F.Shojaei, Ms.Z.Otofat, Ms. F. Pakizekar, and Ms. E. Monem. The authors wish to thank Mr. H.Argasi at the Research Consultation Center (RCC) at Shiraz University of Medical Sciences for his invaluable assistance in editing this manuscript. Also the authors would like to thank Dr. Peyman Jafari at Department of Biostatistics, Shiraz University of Medical Sciences, Shiraz, and Fars, Iran for his precious help for data analysis.

## Conflict of Interest

The authors declare no conflict of interest.

**Table 1:** Details of NIHSS and mNIHSS

**Item number**	**Item name**	**Scoring**
**1A**	LOC	0 = Alert. 1= Not alert 2 = Not alert 3= Responds only with reflex motor or autonomic effects or totally unresponsive, flaccid, and are flexic.
**1B**	LOC questions	0 = Answers both questions correctly. 1 = Answers one question correctly. 2=Answers neither question correctly
**1C**	LOC commands	0 = Performs both tasks correctly. 1 = Performs one task correctly. 2 = Performs neither task correctly.
**2**	Best gaze	0 = Normal. 1 = Partial gaze palsy 2 = Forced deviation
**3**	Visual	0 = No visual loss 1 = Partial hemianopia 2 = Complete hemianopia 3=Bilateral hemianopia ((blind including cortical blindness)
**4**	Facial palsy	0 = Normal 1= Minor paralysis 2 =Partial paralysis 3 = Complete paralysis
**5a**	Left arm motor	0 = No drift 1 = Drift 2 = Some effort against gravity 3 = No effort against gravity 4 = No movement Unknown = Amputation or joint fusion
**5b**	Right arm motor	0 = No drift 1 = Drift 2 = Some effort against gravity 3 = No effort against gravity 4 = No movement Unknown = Amputation or joint fusion
**6a**	Left leg motor	0 = No drift 1 = Drift 2 = Some effort against gravity 3 = No effort against gravity 4 = No movement Unknown = Amputation or joint fusion
**6b**	Right leg motor	0 = No drift 1 = Drift 2 = Some effort against gravity 3 = No effort against gravity 4 = No movement Unknown = Amputation or joint fusion
**7**	Limb Ataxia	0 = Absent. 1 = Present in one limb. 2 = Present in two limbs. Unknown = Amputation or joint fusion
**8**	Sensory	0 = Normal 1= Mild-to-moderate sensory loss 3 = Severe to total sensory loss
**9**	Language	0 = No aphasia 1 = Mild to moderate aphasia 2 = Severe aphasia 3= Mute or global aphasia
**10**	Dysarthria	0 = Normal. 1 = Mild-to-moderate dysarthria 2 = Severe dysarthria Unknown =Intubated or other physical barrier
**11**	Extinction and inattention	0 = No abnormality 1= Visual, tactile, auditory, spatial or personal inattention 2=Profound hemi-inattention or extinction to more than one modality

**LOC:** Level of consciousness

**Table 2:** Cronbach’s Alpha and Convergent Validity for Persian Version of NIHSS and mNIHSS

**Questioner**	**Items**	**Cronbach’s alpha**	**Convergent validity**	
			**Range of correlation** ^a^	**scaling success** ^b^ ** (%)**
**NIHSS**	15	0.81	0.29-0.83	0.8 (80%)
**mNIHSS**	11	0.86	0.49-0.79	1(100%)

**a** Minimum and maximum of correlation between each item and total score. **b** The number of correlations above 0.40/the total number of correlations.
